# *Weissella cibaria* MG5285 and *Lactobacillus reuteri* MG5149 attenuated fat accumulation in adipose and hepatic steatosis in high-fat diet-induced C57BL/6J obese mice

**DOI:** 10.29219/fnr.v65.8087

**Published:** 2021-10-27

**Authors:** Soo-Im Choi, SoHyeon You, SukJin Kim, GaYeong Won, Chang-Ho Kang, Gun-Hee Kim

**Affiliations:** 1 Department of Health Functional Materials, Duksung Women’s University, Seoul, Republic of Korea; 2 R&D Center, MEDIOGEN Co., Ltd., Seoul, Republic of Korea; 3 Department of Food and Nutrition, Duksung Women’s University, Seoul, Republic of Korea

**Keywords:** Limosilactobacillus reuteri MG5149, Weissella cibaria MG5285, anti-obesity, high-fat diet, probiotics

## Abstract

**Background:**

Excessive consumption of dietary fat is closely related to obesity, diabetes, insulin resistance, cardiovascular disease, hypertension, and non-alcoholic fatty liver disease. Recently, probiotics have been highly proposed as biotherapeutic to treat and prevent diseases. Previously, there are studies that demonstrated the beneficial effects of probiotics against metabolic disorders, including obesity and diabetes.

**Objective:**

We investigated the anti-obesity effect and mechanism of action of four human-derived lactic acid bacterial (LAB) strains (*Lacticaseibacillus rhamnosus* MG4502, *Lactobacillus gasseri* MG4524, *Limosilactobacillus reuteri* MG5149, and *Weissella cibaria* MG5285) in high-fat diet (HFD)-induced obese mice.

**Design:**

Obesity was induced in mice over 8 weeks, with a 60% HFD. The four human-derived LAB strains (2 × 10^8^ CFU/mouse) were orally administered to male C57BL/6J mice once daily for 8 weeks. Body weight, liver and adipose tissue (AT) weights, glucose tolerance, and serum biochemistry profiles were determined. After collecting the tissues, histopathological and Western blot analyses were conducted.

**Results:**

Administration of these LAB strains resulted in decreased body weight, liver and AT weights, and glucose tolerance. Serum biochemistry profiles, including triglyceride (TG), total cholesterol, low-density lipoprotein cholesterol, and leptin, pro-inflammatory cytokines, improved. Hepatic steatosis and TG levels in liver tissue were significantly reduced. In addition, the size of adipocytes in epididymal tissue was significantly reduced. In epididymal tissues, *Limosilactobacillus reuteri* MG5149 and *Weissella cibaria* MG5285 groups showed a significantly reduced expression of lipogenic proteins, including peroxisome proliferator-activated receptor γ, CCAAT/enhancer-binding protein α, fatty acid synthase (FAS), and adipocyte-protein 2. In addition, sterol regulatory element-binding protein 1-c and its downstream protein FAS in the liver tissue were significantly decreased. These strains attenuated fat accumulation in the liver and AT by upregulating the phosphorylation of AMP-activated protein kinase and acetyl-CoA carboxylase in HFD-fed mice.

**Conclusion:**

We suggest that *L. reuteri* MG5149 and *W. cibaria* MG5285 could be used as potential probiotic candidates to prevent obesity.

Obesity, a major health problem worldwide, occurs due to an imbalance between energy intake and consumption. Excess fat accumulation in major metabolic tissues is closely related to obesity, diabetes, insulin resistance, cardiovascular disease, hypertension, and non-alcoholic fatty liver disease (NAFLD) (1, 2).

There are approximately 500–1,000 intestinal microflora species, and the changes in these gut microbiota are closely related to obesity and metabolic disorders (3). Bacteroides and Firmicutes are the two predominant phyla, accounting for over 90% of healthy gut microbes (4). Intestinal microbes, in particular, regulate lipid and glucose metabolism, and affect energy extraction, inflammation, and secretion of neuroendocrine peptides (5).

Probiotics are living, non-pathogenic microorganisms, in which symbiosis with intestinal microorganisms has a beneficial effect on the health of the host (6). Lactic acid bacteria (LAB) have been used as major probiotic ingredients, and *Lactobacillus* and *Bifidobacterium* are currently the most common bacteria used as probiotics that improve gastrointestinal disorders and inhibit the excessive growth of pathogenic intestinal bacteria (7). Probiotics play an important role in metabolic homeostasis by influencing the composition of microorganisms in the intestine, improving internal health, and restoring the microbial migration properties of obesity (8, 9). Some probiotics have been found to induce anti-obesity effects by regulating lipid and glucose metabolism (10). Recently, several therapeutic platforms to treat and prevent diseases using probiotics have been proposed. In addition, evidence has shown the beneficial effects of probiotics against metabolic disorders, including obesity and diabetes (11).

In the previous study, four strains (*Lacticaseibacillus rhamnosus* MG4502, *Lactobacillus gasseri* MG4524, *Limosilactobacillus reuteri* MG5149, and *Weissella cibaria* MG5285) isolated from infant feces showed higher antioxidant activity, reduced lipid accumulation, and inhibition of α-glucosidase activity. In addition, probiotic properties based on acid resistance, tolerability, hemolysis, auto-aggregation, antibiotic susceptibility, enzyme production, and biochemical profiles were confirmed. Therefore, the purpose of this study was to determine whether these four selected strains affect the fat accumulation in liver and adipose tissue (AT) and serum biochemical changes in a high-fat diet (HFD)-induced obese mouse model, and to clarify the mechanism of action.

## Materials and methods

### Materials

Antibodies against peroxisome proliferator-activated receptor gamma (PPARγ), CCAAT or enhancer-binding protein alpha (C/EBPα), fatty acid synthase (FAS), adipocyte protein 2 (aP2), acetyl-CoA carboxylase (ACC), phosphorylated ACC (p-ACC), Sterol regulatory element-binding proteins (SREBPs), AMP-activated protein kinase (AMPK), phosphorylated AMPK (p-AMPK), and β-actin were purchased from Cell Signaling Technology (Danvers, MA, USA). All other reagents were purchased from Sigma-Aldrich (St. Louis, MO, USA).

### Preparation of LAB strains

The four LAB strains (MG4502, MG4524, MG5149, and MG5285) used in this study were supplied by MEDIOGEN Co., Ltd (Jecheon, Korea). To powder the LAB strains, the newly cultured bacterial pellet was mixed well with a cryoprotectant at a ratio of 1:25 (w/w). Each bacterial cell suspension was dispersed in wall materials and freeze-dried (Heto Drywinner, Allerod, Denmark) (12). The powdered cells were harvested and stored at 4°C until further use.

### Animals and experimental design

Male C57BL/6J mice (4-week-old, 20 ± 2.0 g) were obtained from Orientbio Co., Ltd (Seongnam, Korea). All mice were housed under controlled environmental conditions (temperature 23 ± 1.0°C, 45 ± 5% RH, and 12 h light–dark cycle) with free access to food and water. All mice were treated according to the Guide for the Care and Use of Laboratory Animals (Institute of Laboratory Animal Resources, Commission on Life Sciences, National Research Council, 1996). The experimental protocols were approved by the Institutional Animal Care and Use Committee of Duksung Women’s University (Approval no. 2020-001-001), and all efforts were made to minimize suffering. To induce obesity, following acclimatization for a week, the experimental groups were randomly divided into six groups as follows: normal diet (ND), high-fat diet (HFD), HFD + *Lacticaseibacillus rhamnosus* MG4502 (MG4502), HFD + *Lactobacillus gasseri* MG4524 (MG4524), HFD + *Limosilactobacillus reuteri* MG5149 (MG5149), and HFD + *Weissella cibaria* MG5285 (MG5285). The ND group fed a normal diet containing 16% kcal fat, 20% kcal protein, and 64% kcal carbohydrate, 3.76 kcal/g (D12492, Research, NJ, USA) and an HFD group and strain-treated groups were fed an HFD with 60% kcal fat, 20% kcal protein, and 20% kcal carbohydrate, 5.21 kcal/g (D12492, Research, NJ, USA) for 8 weeks. Each strain (2 × 10^8^ CFU/mouse) powder was sufficiently dissolved in sterilized distilled water (0.1 mL/10 g b.w./mouse) and orally administered using a stainless feeding needle of 18–20 g once a day at 10 am. The dosage of the strain was derived from an *in vitro* preliminary study and previous studies in similar HFD-induced obese mice models (13).

### Body weight and food intake

The body weight and food intake of all mice were measured weekly. Food intake was calculated as the amount of food remaining in the cage from the food served simultaneously, and the food efficacy ratio (FER) was expressed as the ratio of food intake divided by the average weight of the mice.

### Oral glucose tolerance test

The oral glucose tolerance test (OGTT) was performed after a treatment period of 8 weeks. Mice were fasted for at least 12 h, and glucose (2 g/kg b.w.) was orally administered. Thereafter, blood glucose levels were measured at 15, 30, 60, 90, and 120 min by tail vein puncture using a glucose meter (14).

### Weight of liver and AT

At the end of the treatment period, mice were fasted overnight, and all mice were anesthetized by CO^2^ inhalation and euthanized by cardiac puncture. The liver and epididymal (EP), subcutaneous (SubQ), and mesenteric AT were rapidly dissected, rinsed with phosphate-buffered saline, weighed, and visually inspected. All liver and EP AT were frozen in liquid nitrogen and stored at **−**70°C until analysis.

### Serum biochemical analysis

Each blood sample collected from mice was left at room temperature for at least 30 min, and the serum was recovered by centrifugation at 3,000 rpm for 20 min at 4°C. Serum levels of alanine transaminase (ALT), aspartate transaminase (AST), triglycerides (TG), total cholesterol (TCH), low-density lipoprotein cholesterol (LDL-c), high-density lipoprotein cholesterol (HDL-c), and glucose were measured using a biochemical analyzer (Hitachi Ltd, Japan). Plasma leptin and adiponectin concentrations were quantified using a commercial ELISA kit (R&D System, Minneapolis, MN, USA).

### Liver TG content

The liver tissue (100 mg) was homogenized by adding 1 mL of 5% Nonidet P-40 substitute to extract the lipids. The TG content in the lipid extracts was measured using a commercial quantification kit (Sigma-Aldrich, St. Louis, MO, USA). The measured value was quantified using the protein concentration of the lipid extracts measured by the Bradford assay.

### Histopathological analysis

To evaluate the effects of LAB on steatosis and lipogenesis, histopathological changes in the liver and AT were analyzed. Briefly, the fixed tissue in 10% formalin was subjected to tissue processing, such as cutting, dehydration, and hematoxylin and eosin (H&E) staining. Histological changes in liver tissue were scored for steatosis and inflammation, according to the method of Liang et al. (2014) (15). Steatosis was evaluated as the sum of the scores of macrovesicular steatosis and microvesicular steatosis, respectively. Severity is expressed as follows: 0 (<5%), 1 (5–33%), 2 (34–66%), and 3 (66–100%, coverage), depending on the percentage of total area affected categories. Inflammation was assessed by counting the number of inflammatory foci per field in five different fields and scored as follows: 0 (<0.5 foci), 1 (0.5–1.0 foci), 2 (1.0–2.0 foci), and 3 (> 2.0 foci). Images were obtained using an optical microscope (Olympus BX53, Japan) at a magnification of 100×. All histological procedures were performed by a board-certified toxicological pathologist in a blinded manner.

### Western blot analysis

The expression of lipogenic proteins in the liver and AT was assessed by Western blot analysis. Briefly, tissues were lysed with RIPA buffer and centrifuged at 13,000 rpm for 20 min at 4°C. Protein levels in lysates were quantified by the Bradford assay (Bio-Rad, Hercules, CA, USA). Protein (20 μg) was mixed with 10% SDS loading buffer, electrophoresed on a 10% SDS–polyacrylamide gel, transferred to a nitrocellulose membrane, and then blocked with 5% skim milk in Tris-buffered saline with Tween-20 (TBST) for 90 min. Membranes were incubated overnight at 4°C with primary antibodies against PPARγ, C/EBPα, FAS, aP2, p-ACC, ACC, p-AMPK, AMPK (1: 1,000), or β-actin (1: 3,000). After washing three times with TBST buffer, the membranes were incubated with a goat anti-rabbit IgG-HRP conjugated secondary antibody (1: 3,000) at room temperature for 90 min and washed three times with TBST buffer. Specific proteins were visualized using an ECL detection kit (Thermo-Fisher Scientific Inc., Cambridge, MA, USA) and quantified using the Image J program (National Institutes of Health, Bethesda, MD, USA). The expression level of the proteins was normalized to that of β-actin.

### Statistical analysis

All data are expressed as the mean ± standard deviation (SD) of triplicate experiments. Statistical analysis was performed by one-way analysis of variance (ANOVA) using SPSS version 22 (IBM Corp., NY, USA). The significance between groups was analyzed using Duncan’s multiple range test, and statistical significance was set at P < 0.05.

## Results

### Change of body weight and food intake

**Fig. 1:**
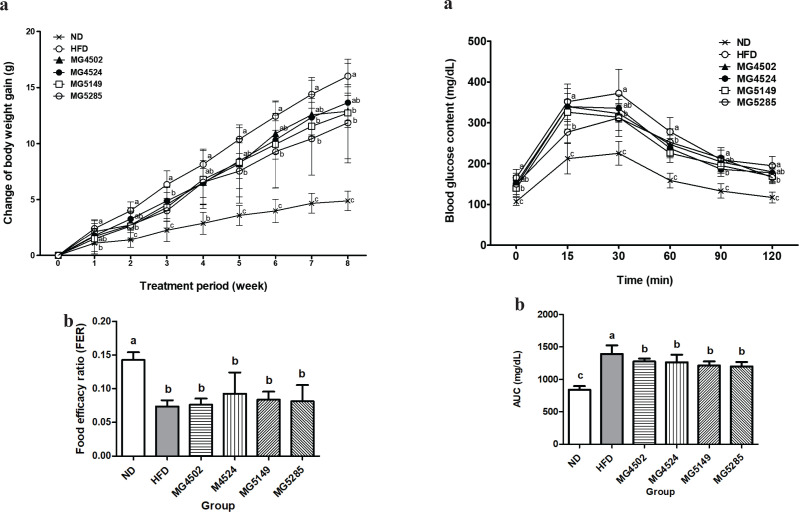
Changes in body weight and food intake in HFD-induced obese mice. Body weight and food intake were measured weekly. ND: normal diet, HFD: high-fat diet. Results are presented as the mean ± SD (*n* = 6). Different letters indicate significant differences between means at *p* < 0.05 by Duncan’s multiple range test.

To evaluate the anti-obesity effects of the four candidate strains (MG4502, MG4524, MG5149, and MG5285), changes in body weight and food intake were measured (Fig. 1). After 8 weeks, the body weight of the HFD group was significantly increased (16.02 ± 1.52 g), which was approximately 3.3 times higher than that of the ND group (4.88 ± 0.87 g). In this study, HFD-induced obese mice were established successfully. All LAB-administered groups had a significantly reduced body weight compared with HFD groups by 26% for MG5285 (11.85 ± 3.22 g),> 20.5% for MG5149 (12.73 ± 4.45 g), > 19.6% for MG4502 (12.88 ± 1.43 g), and > 14.8% for MG4524 (13.65 g ± 1.61 g).

The food intake of the ND and HFD groups were estimated to be 3.3 ± 0.24 g/day and 2.31 ± 0.35 g/day, respectively, and the food intake of LAB-administered groups was estimated to be in the range of 2.08–2.39 g/day. The FER was 0.14 in the ND group, which was significantly different from that in the HFD group. However, there was no significant difference between the HFD-treated groups. Therefore, in HFD-induced mice, the administration of these four LAB strains effectively reduced the body weight without any difference in the efficiency of dietary intake.

### Effect of LAB strains on glucose tolerance

**Fig. 2:**
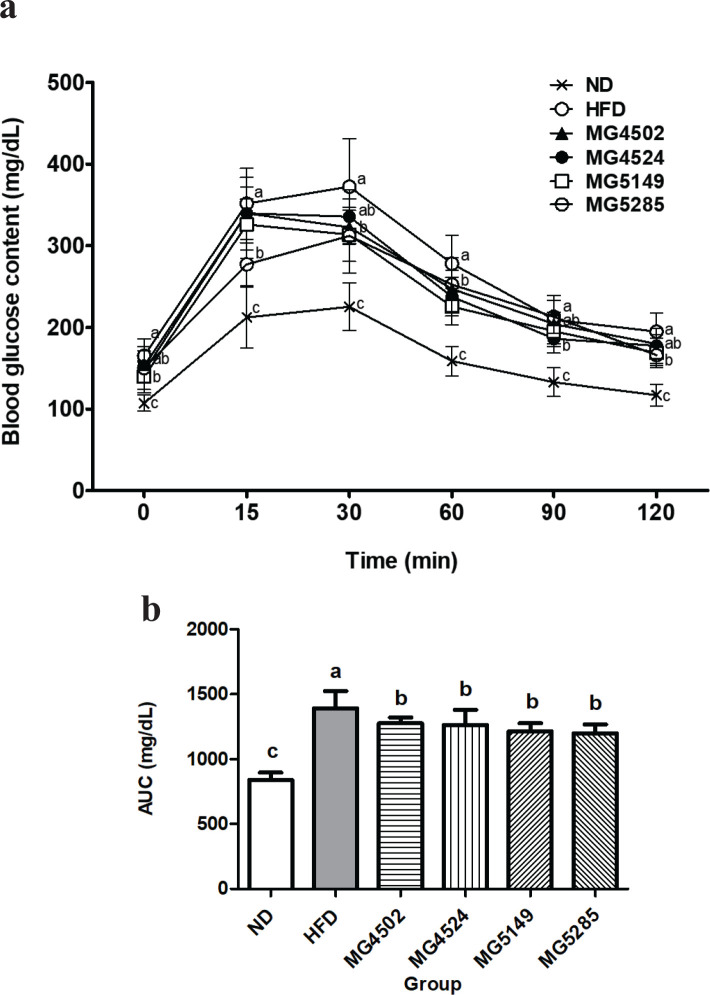
Glucose tolerance in HFD-induced obese mice. Glucose tolerance was determined by measuring blood glucose levels and area under curve (AUC) value using the oral glucose tolerance test (OGTT) after the 8-week treatment period. ND: normal diet, HFD: high-fat diet. Results are presented as the mean ± SD (*n* = 6). Different letters indicate significant differences between means at *p* < 0.05 by Duncan’s multiple range test.

Eight weeks after HFD supply, an OGTT was performed to confirm the effects of the candidate strains on glucose tolerance. OGTT was performed using blood obtained from the tail vein at 0, 15, 30, 60, 90, and 120 min after glucose administration (Fig. 2A). At 30 min, the blood glucose levels in the ND and HFD groups increased to 118.25 mg/dL and 207.13 mg/dL, respectively, compared with the fasting blood glucose level, whereas in all LAB-administered groups, the blood glucose levels were lower than that in the HFD group. In particular, the blood glucose levels in the MG5285 group increased to 161.88 mg/dL, which was the lowest among all the LAB-administered groups. After 120 min, the blood glucose level in the ND group recovered to levels similar to those of fasting blood glucose. The blood glucose levels in the MG4524 group (168.8 ± 16.61 mg/dL) and the MG5285 group (166.0 ± 14.42 mg/dL) were significantly reduced compared with those in the HFD group (194.9 ± 22.77 mg/dL). The glucose levels in the rest of the groups remained at 30 mg/dL, which was higher than the initial fasting level. The area under curve (AUC) of all the treated groups was calculated (Fig. 2B). The AUC of the ND group was 840.75 ± 55.33 mg/dL, whereas that of the HFD group was 1391.7 ± 132.00 mg/dL, which was 1.7-fold higher than that of the ND group (840.8 ± 55.33 mg/dL). However, compared with the HFD group, the blood glucose levels in all LAB-administered groups were significantly lower, with an average of 1238.81 mg/dL. The MG5285 group showed the lowest levels with a 14% decrease.

### Changes of liver and AT weights

The degree of fat accumulation in the liver and AT (EP, SubQ, and mesenteric) eight weeks after the HFD supply was measured as tissue weight (Table 1). The liver weight was significantly higher by 160 mg in the HFD group than that in the ND group. Meanwhile, compared with the HFD group, the liver weight of the LAB-administered groups was reduced by 19.1% with an average of 0.92 g; however, there was no significant difference between the LAB-administered groups. The EP AT weight was increased five times in the HFD group compared with that in the ND group, and the EP AT weight in the MG5149 and MG5285 groups was significantly lower than that in the HFD group.

**Table 1:** Weight of the liver and adipose tissues of HFD-induced obese mice

Groups		Treatment (CFU/mouse)	Liver	Adipose tissue (AT) weight (g)			Total AT weight (g)
				EP^3^	SubQ^4^	Mesenteric	
ND^1^		–	0.99 ± 0.06^b^	0.41 ± 0.06^c^	0.30 ± 0.07^c^	0.37 ± 0.10^d^	1.08 ± 0.19^c^
HFD^2^		–	1.14 ± 0.13^a^	2.11 ± 0.31^a^	2.6 ± 0.40^a^	1.13 ± 0.06^a^	5.83 ± 0.63^a^
HFD +	MG4502	2 × 10^8^	0.93 ± 0.08^b^	1.94 ± 0.31^ab^	1.23 ± 0.26^b^	0.84 ± 0.20^bc^	4.02 ± 0.47^b^
	MG4524		0.95 ± 0.16^b^	1.85 ± 0.35^ab^	1.30 ± 0.45^b^	1.00 ± 0.18^ab^	4.16 ± 0.79^b^
	MG5149		0.93 ± 0.16^b^	1.66 ± 0.42^b^	1.25 ± 0.52^b^	0.80 ± 0.19^c^	3.70 ± 0.99^b^
	MG5285		0.90 ± 0.11^b^	1.61 ± 0.29^b^	1.25 ± 0.38^b^	0.79 ± 0.17^c^	3.65 ± 0.63^b^

1 ND: normal-diet

2 HFD: high-fat-diet

3 EP AT: epididymal AT

4 SubQ AT: subcutaneous AT. The total AT weight was calculated as the sum of the EP, subQ, and mesenteric AT weights. Results are presented as the mean ± SD (*n* = 6). Different letters indicate significant differences between means at *p* < 0.05 by Duncan’s multiple range test.

The subQ AT weight increased eight-fold in the HFD group but decreased by 50% in all LAB-administered groups. In particular, in the MG5285 group, mesenteric AT weights were significantly reduced compared with those in the HFD group. The total AT weight was calculated as the sum of the EP, subQ, and mesenteric AT weights, excluding the liver. The total AT weight in the HFD group was five times higher than that in the ND group. In contrast, the AT weight was reduced by more than 28% following LAB administration. These results revealed that the administration of these LAB effectively suppressed fat accumulation in the liver and AT caused by HFD.

### Effect of LAB strains on serum profiles

Eight weeks after HFD supply, biochemical changes in the blood due to HFD supplementation were confirmed by serum analysis (Table 2). The HFD group had significantly higher levels of all parameters than the ND group; however, all LAB-administered groups showed significant differences compared with the HFD group. The ALT level in the LAB-administered groups significantly decreased to a similar degree as that in the ND group, averaging approximately 37.4% lower than that in the HFD group. Compared with those in the HFD group, the AST levels in the three LAB-administered groups (MG4502, MG5149, and MG5285) were significantly reduced, with an average of 169.18 IU/L. However, there was no significant difference in the MG4524 group compared with the HFD group.

**Table 2:** Biochemical analysis of plasma in HFD-induced obese mice

Profile	Group					
	ND^1^	HFD^2^	HFD + LAB strain (2 × 10^8^ CFU/mouse)			
			MG4502	MG4524	MG5149	MG5285
AST (IU/L)	134.0 5 ± 73.85^c^	384.7 ± 81.47^a^	186.93 ± 80.96^b^	51.40 ± 17.94^b^	54.60 ± 29.14^b^	49.86 ± 13.11^b^
ALT (IU/L)	37.35 ± 10.27^b^	81.95 ± 21.18^a^	49.30 ± 9.75^b^	51.40 ± 17.94^b^	54.60 ± 29.14^b^	49.86 ± 13.11^b^
TG (mg/dL)^3^	12.19 ± 2.96^d^	62.16 ± 15.39^a^	44.44 ± 12.20^b^	38.58 ± 6.57^bc^	30.39 ± 14.04^c^	30.00 ± 10.29^c^
TCH (mg/dL)^4^	107.79 ± 6.37^c^	172.4 ± 21.56^a^	150.75 ± 7.54^b^	143.16 ± 16.94^b^	143.80 ± 21.34^b^	138.11 ± 12.29^b^
HDL-c (mg/dL)	54.54 ± 3.54^c^	67.09 ± 3.17^a^	65.65 ± 2.55^ab^	61.70 ± 4.72^b^	62.73 ± 4.25^b^	63.50 ± 3.72^ab^
LDL-c (mg/dL)	4.86 ± 0.81^e^	10.70 ± 1.88^a^	8.16 ± 0.93^bc^	8.51 ± 1.58^b^	6.95 ± 1.49^cd^	6.48 ± 1.17^d^
HDL-c/TCH (%)	50.70 ± 3.21^a^	39.42 ± 5.01^c^	43.65 ± 2.92^b^	43.33 ± 2.65^bc^	44.12 ± 4.18^b^	46.27 ± 4.69^b^
Glucose (mg/dL)	247.84 ± 23.54^bcd^	303.83 ± 43.64^a^	275.33 ± 14.13^ab^	255.77 ± 29.01^abc^	240.04 ± 29.34^cd^	228.67 ± 25.82^d^
Leptin (ng/mL)	3.85 ± 1.44^c^	52.00 ± 4.83^a^	31.08 ± 5.19^b^	34.61 ± 8.69^b^	28.90 ± 8.88^b^	29.59 ± 6.33^b^
Adiponectin (μg/mL)	152.70 ± 14.89^a^	93.42 ± 7.85^d^	123.7 ± 17.01^bc^	112.5 ± 17.01^c^	127.8 ± 12.04^b^	135.10 ± 11.51^b^

1 ND: normal-diet

2 HFD: high-fat-diet

3 TG: triglyceride

4 TCH: total cholesterol. Results are presented as the mean ± SD (*n* = 6). Different letters indicate significant differences between means at *p* < 0.05 by Duncan’s multiple range test.

The TG level was approximately five-fold higher in the HFD group than that in the ND group, while TG levels in the MG5149 and MG5285 groups were reduced by approximately 50% or more compared with those in the HFD group. In addition, serum TCH, LDL-c, and HDL-c levels were significantly higher in the HFD group than in the ND group. LDL-c levels were reduced by approximately 35 and 39% in the MG5149 and MG5285 groups compared with those in the HFD group. The percentage of HDL-c/TCH was reduced by 22% in the HFD group compared with the ND group, but was increased by 12 and 17% in the MG5149 and MG5285 groups compared with the HFD group. Glucose levels were significantly reduced in the MG5149 and MG5285 groups compared with those in the HFD group. These results revealed that the MG5149 and MG5285 groups showed significant reductions in all serum parameters compared with the HFD group.

The serum leptin level in the HFD group was significantly higher than that in the ND group. In contrast, in all the LAB-administered groups, the average serum leptin levels decreased by 40.3% compared with those in the HFD group. The MG5149 and MG585 groups showed the highest decreases in serum leptin levels compared with the HFD group. In addition, the serum adiponectin level in the ND group decreased by approximately 40% compared with that in the HFD group. However, the reduced adiponectin level was significantly increased after LAB administration. In particular, the MG5285 group showed the highest levels of adiponectin.

### Effect of LAB strains on histopathological changes in liver and AT

**Fig. 3:**
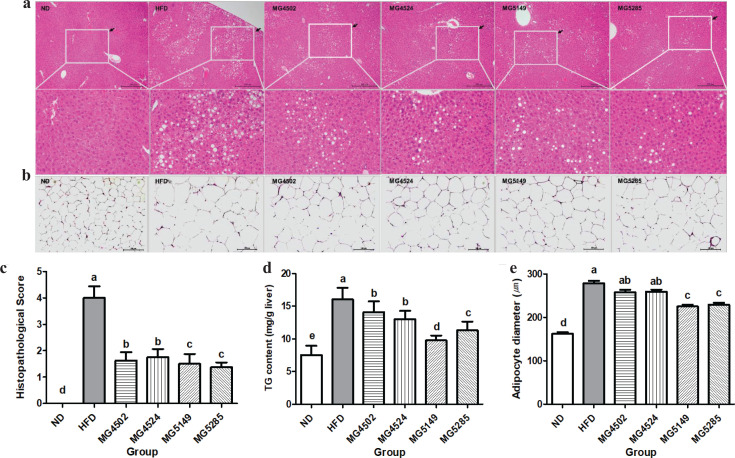
Histopathological changes in the liver and epididymal adipose tissue (AT) of HFD-induced obese mice. Representative photographs of hematoxylin and eosin (H&E)-stained sections (100 × magnification) of (A) liver tissue, (B) epididymal AT, (C) histopathological score, (D) triglyceride (TG) content, and (E) adipocyte diameter. The white square in the figure is an enlarged area where steatosis was induced. ND: normal diet, HFD: high-fat diet. Results are presented as the mean ± SD (*n* = 6). Different letters indicate significant differences between means at *p* < 0.05 by Duncan’s multiple range test.

Histopathological changes in the H&E-stained liver and EP AT are shown in Fig. 3A-B. Hepatic steatosis was clearly identified as the main lesion in the HFD group. Macrovesicular steatosis was mainly observed rather than microvesicular steatosis. However, hypertrophy and inflammation were marginally induced in all HFD fed groups. The histopathological score for hepatic steatosis and inflammation in the HFD group was 4.0 ± 1.15 (Fig. 3C). However, the level of liver tissue changes in the LAB-administered groups was significantly decreased compared with that in the HFD group. The scores of the MG5149 (1.50 ± 1.07) and MG5285 (1.375 ± 0.52) groups were significantly lower than those of the MG4502 (1.625 ± 0.92) and MG4524 (1.625 ± 0.74) groups. In addition, the TG content in liver tissues of the HFD group (16.05 ± 1.76mg/g liver) was significantly 2.14 times higher than that in the ND group (7.51 ± 1.43 mg/g liver). All LAB-administered groups showed a reduction in the liver TG content. In particular, the liver TG content in the MG5149 group (9.76 ± 0.75 mg/g liver) was reduced by 39.2% than in the HFD group (Fig. 3D).

Histological analysis of epididymal AT showed that the size of adipocytes was significantly increased in the HFD group (278.655 ± 15.12 μm) compared with that in the ND group (162.07 ± 11.29 μm). However, in the MG5149 (225.041 ± 12.58 μm) and MG5285 (229.126 ± 12.83 μm) groups, the fat cell diameter was significantly decreased compared with the HFD group (*p* < 0.001; Fig. 3B and E).

### Effect of LAB strains on lipogenic proteins expression in EP AT and liver tissue

**Fig. 4:**
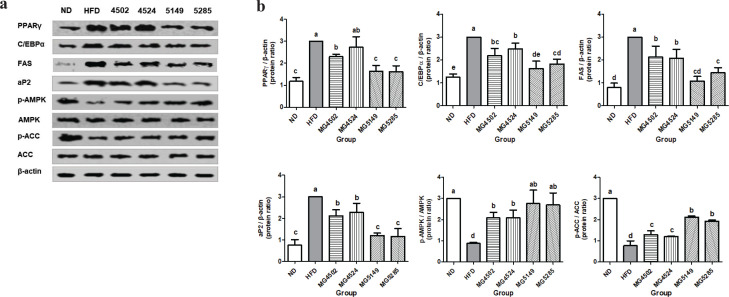
Lipogenic protein expression in epididymal adipose tissue (AT) of HFD-induced obese mice. ND: normal diet, HFD: high-fat diet. Following 8-weeks administration of LAB strains, protein expression in epididymal AT was analyzed by western blot analysis. (A) western blot image and (B) relative protein levels of PPAR-γ, CEBP/α, FAS, aP2, LPL, p-AMPK/AMPK, and p-ACC/ACC. Results are presented as the mean ± SD (*n* = 6). Different letters indicate significant differences between means at *p* < 0.05 by Duncan’s multiple range test.

The expression of lipogenic proteins in the liver and EP AT was measured by Western blotting. The expression levels of PPARγ, C/EBPα, FAS, aP2, p-AMPK, AMPK, p-ACC, and ACC in epididymal AT were normalized to β-actin levels (Fig. 4A–B). The expression levels of PPARγ and aP2 were significantly increased in the HFD group. However, the expression levels of PPARγ and aP2 in the MG5149 and MG5285 groups were significantly lower by 45.7 and 60.7%, respectively, than those in the HFD group. The expression levels of C/EBPα and FAS in the MG5149 group were reduced by 45.8 and 63.9%, respectively, compared with those in the HFD group. The MG5149 group showed the most reduced levels of C/EBPα and FAS among all the LAB-administered groups. In addition, the LAB administration group showed significantly increased levels of phosphorylated AMPK and ACC. In particular, the expression levels of p-AMPK and p-ACC in the MG5149 and MG5285 groups were 3.1 and 2.8 times higher, respectively, than that in the HFD group.

**Fig. 5:**
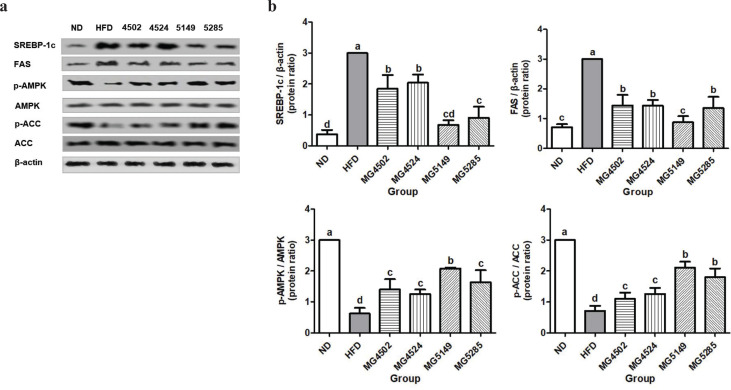
Expression of proteins associated with lipid metabolism in liver tissue in liver tissue of HFD-induced obese mice. ND: normal diet, HFD: high-fat diet. Following 8-weeks of LAB strains administration, protein expression in liver tissue was analyzed by western blot analysis. (A) western blot image and (B) relative protein levels of SREBP-1c, FAS, p-AMPK/AMPK, and p-ACC/ACC. Results are presented as the mean ± SD (*n* = 6). Different letters indicate significant differences between means at *p* < 0.05 by Duncan’s multiple range test.

SREBP-1c and FAS expression levels in liver tissue were significantly higher in the HFD group (Fig. 5A–B). The expression levels of p-AMPK and p-ACC were normalized to those of AMPK and ACC, respectively. p-AMPK and p-ACC were significantly increased in the strain-administered groups compared with the HFD group, which was significant in the MG5149- and MG5285-administered groups.

## Discussion

Trillions of microbes reside in the human body. The intestinal microbiota influences the metabolism of the host metabolism; they regulate the immune system, digestion, absorption, and synthesis. Several studies have revealed that dysbiosis of the intestinal microbiota, which is an imbalance of microbial communities, disrupts the host’s energy metabolism, and contributes to obesity and metabolic syndrome (16, 17). Dietary habits and nutrition are very influential in maintaining intestinal health and can be useful or toxic to the host. Therefore, the action of microbes in the intestine is crucial to healthy body. In particular, dietary fat affects the growth and survival of the intestinal microflora (18), and the human body acts differently when used to harvest, storage, and utilization of energy intake depending on the composition of microflora (16). To date, many studies have shown the beneficial effects of probiotics on human health. For example, *Lactobacillus* spp. could improve NAFLD, and *Lactobacillus* spp. and *Bifidobacterium* spp. exert anti-obesity effects by controlling energy metabolism (19, 20). In this study, we showed the effects of four *Lactobacillus* strains on HFD-induced mice based on the screening of *Lactobacillus* strains that showed anti-adipogenic effects in 3T3-L1 cells.

Body weight is a primary indicator of obesity; thus, body weight change could signify the inhibition of obesity (4). The intake of four *Lactobacillus* strains reduced the HFD-induced weight gain. The food intake in all the groups was not significantly different, which means that the groups that consumed HFD gained weight due to the intake of dense calories. A previous study elucidated that the regular intake of probiotics effectively inhibited weight gain in an HFD-induced animal model (21). In this study, supplementation with MG5149 and MG5285 significantly prevented weight gain. Wang et al. (22) compared the anti-obesity effects of several types of LAB using a mouse model and confirmed that none of the species were effective in reducing weight. With regard to the result, Wang et al. (22) reported that each strain has selective effects on obesity and metabolic diseases.

HFD-induced obesity has been shown to improve glucose tolerance (23). In particular, increased visceral and liver fat are closely related to impaired glucose tolerance. Moreover, when impaired glucose tolerance is aggravated, it develops into type-2 diabetes (24). Foley et al. (25) evaluated blood glucose levels in HFD-induced mice and confirmed that only 4 days of HFD consistently increased blood glucose levels for 100 min. In this study, glucose intolerance was confirmed in HFD-induced obese mice. However, four LAB strains significantly reduced blood glucose levels, especially in the *W. cibaria*-administered group, which substantially improved glucose metabolism. Lim et al. (26) additionally administered *L. sakei* to mice fed an HFD for 4 weeks and confirmed that the glucose levels of mice improved, and insulin levels also recovered to normal levels. Furthermore, in a study by Eslamparast et al. (27), the administration of probiotics to diabetic rats ameliorated increased blood glucose levels in an insulin-independent manner. Foley et al. (25) reported that the long-term administration of an HFD might alter the intestinal microbiota and affect the postprandial glucose response. In this study, the administration of four LAB strains may help improve the intestinal environment, which was affected by HFD, and activated glucose metabolism.

Adipose tissue is distributed in many parts of the body and is involved in various metabolic processes, such as hormone, fat, and energy metabolism. The excessive intake of fat causes hyperplasia and hypertrophy of adipocytes in the white AT, which leads to lipid accumulation in the tissue (28). In addition, in the lipid metabolism process, surplus body fat produces free fatty acids, which moves through the portal vein, augmenting liver fat deposition (24). In this study, after 8 weeks of HFD intake, fat accumulated in the tissues as the weight of the liver and AT (epididymal, mesenteric, retroperitoneal, perirenal, and subcutaneous) increased. However, the intake of four LAB strains inhibited lipid accumulation in the liver and AT. These results indicate that *Lactobacillus* intake positively affects lipid metabolism.

The liver is involved in glucose, fatty acid, and energy metabolism and is an important organ responsible for detoxification (29). Abnormalities in peripheral metabolism due to liver obesity can cause fat to flow to the liver tissue and induce stress by inducing lipid accumulation (30). The hepatic enzymes, ALT and AST are clinical indicators of inflammation and liver destruction (4). Eight weeks of HFD intake increased ALT and AST levels in serum. Furthermore, the levels of the indicators related to lipid metabolism, such as TG, LDL, and TCH, increased. In contrast, the administration of four LAB strains improved liver function and lipid metabolism by reversing these results. The findings of Xie et al. (31) were consistent with the results of this study that the ingestion of *Lactobacillus* and *Bifidobacterium* improved TCH, TG, and LDL levels, as well as improved lipid metabolism, in the serum of obese rats.

Leptin and adiponectin are adipokines released from AT (32), which maintain glucose, lipid, and energy homeostasis. In response to dietary fat increases, leptin levels increase in the serum. Adiponectin, however, promotes fatty acid oxidation and peripheral insulin sensitivity (33, 34). In this study, the intake of four LAB strains normalized leptin and adiponectin levels in the serum of obese mice. In a previous study, the *L. rhamnosus* GG (*L.* GG) administration to mice increased epididymal AT deposition and serum adiponectin levels (34).

It has been reported that a chronic high-calorie diet could induce fat accumulation and produce lipid droplets in liver tissue leading to NAFLD (28), which is characterized by steatosis and inflammation in the liver. Due to impaired fatty acid metabolism caused by excessive intake of dietary fat, lipids gradually accumulate in the liver and each AT in the form of TGs. Accumulated TGs in the body eventually causes steatosis and inflammation in hepatocytes, resulting in NAFLD (35, 36). Multiple strains of probiotics, such as *Streptococcus thermophilus* and several *Lactobacillus* and *Bifidobacteria*, positively affected NAFLD by inhibiting the secretion of inflammatory cytokines in HFD-induced rats (37). Yen et al. (1) demonstrated that AST and ALT levels were normalized by administration of *B. lactis* V9 to obese mice for 4 weeks, while Zhao et al. (4) reported that *L. plantarum* CQPC02 exerts a hepato-protective effect by reducing the accumulation of lipid droplets and hepatic steatosis. In this study, four LAB strains reduced the TG content, hepatocyte hypertrophy, and lipid droplet size. Moreover, they attenuated AST and ALT levels in the serum, showing results similar to those of the previous studies. In particular, the MG5149 and MG5285 effectively improved the serum biochemical profiles in HFD-induced obese mice. These results indicate that all LAB strains used in this study have a beneficial effect on HFD-induced NAFLD.

Lipogenesis and lipolysis are required in AT to maintain and control energy homeostasis. This lipid metabolic system is controlled by several factors, such as leptin, adiponectin, and energy consumption (38). However, altered lipid metabolism causes an increase in the number of adipocytes, hyperplasticity, or enlargement of adipocytes. The activation of adipogenesis promotes lipid accumulation (39). PPARγ and C/EBPα are transcription factors involved in adipogenesis that regulate lipid metabolism. These lipid metabolic mediators play a critical role in lipid synthesis and lipogenesis via synergistic effects (4). As expected, the administration of four LAB strains attenuated the expression of PPARγ and C/EBPα, and inhibited the synthesis of the lipogenic enzymes, FAS and aP2. In particular, *L. reuteri* and *W. cibaria* more effectively reduced the expression of adipogenic factors and enzymes. These results suggest that *L. reuteri* and *W. cibaria* regulate adipogenic transcription factors in adipocyte differentiation programs (40). SREBPs are predominantly expressed in the liver and AT, and are responsible for fatty acid and cholesterol metabolism (30). It has been reported that SREBP-1c stimulates AT by activating hepatic *de novo* lipogenesis. *De novo* lipogenesis mainly occurs in the liver rather than in AT and is closely related to NAFLD (38).

AMPK is a cellular energy sensor that regulates energy steatosis. AMPK serves as a cue for ATP production for the hormonal and nutritional signals of AT, thereby achieving stable hormonal and lipid metabolism. When AMPK is inactivated, ACC, one of the downstream factors, is dephosphorylated. Dephosphorylated ACC catalyzes the conversion of acetyl-CoA into malonyl-CoA, leading to *de novo* lipogenesis (41). Therefore, any complication with AMPK activation affects the expressions of genes and proteins associated with lipid metabolism (29). In this study, AMPK and ACC expression levels in the HFD group were reduced; however, the administration of four LAB strains led to the phosphorylation of AMPK and ACC. In particular, the administration of *L. reuteri* and *W. cibaria* induced the most pronounced phosphorylation of AMPK and ACC, which is consistent with the expression of lipogenesis-related proteins, such as PPARγ, C/EBPα, and SREBP-1c. These results reveal that the lipogenesis-inhibitory effect of these LAB strains is regulated by the AMPK pathway. Hossain et al. (42) confirmed that *L. rhamnosus* DS0508 promotes thermogenesis, resulting in lipolysis in both *in vitro* and *in vivo* models. Additionally, Kadooka et al. (43) demonstrated that *L. gasseri* SBT2055 administration could reduce abdominal obesity in adults, while Qiao et al. (44) reported that *L. reuteri* L3 controlled PPARγ expression and its downstream pathway, resulting in anti-obesity effects.

*W. cibaria* MG5285 showed similar or better anti-obesity effects than the other three LAB strains used in this study. *Weissella* is a relatively recently known LAB genus, consisting of 23 species, many of which exist in fermented foods, such as kimchi. They ferment glucose through a hetero-lactate fermentation pathway (45). Among the *Weissella* genus, *W. cibaria* is the most prevalent species in kimchi and has been reported to have potential functional effects, such as antioxidant and anti-inflammatory effects (46). Despite the various benefits of *W. cibaria*, there are relatively few reports on its anti-obesity and hepato-protective roles. Therefore, this study suggests that *W. cibaria* may be a potential candidate for developing an anti-obesity functional food source.

## Conclusion

This study demonstrated that the oral administration of LAB (*L. rhamnosus* MG4502, *L. gasseri* MG4524, *L. reuteri* MG5149, and *W. cibaria* MG5285) prevented body weight gain, improved glucose tolerance, and lipid metabolism, thereby preventing fatty liver disease in obese mice. Moreover, low lipogenic protein expression was observed in the liver and AT. Among the four LAB strains, *W. cibaria* showed the most effective inhibition of obesity by regulating the AMPK/ACC pathway. Hence, the study results offer significant insights into the anti-obesity effect of *W. cibaria* as a functional food source.

## Funding

This research work was financially supported by the Ministry of Small and Medium-sized Enterprises (SMEs) and Startups (MSS), Korea, under the ‘Regional Enterprise Open-Innovative Voucher Program (R&D, Project P0010724)’ supervised by the Korea Institute for Advancement of Technology (KIAT).

## Author contributions

Designed experiments: Soo-Im Choi, Chang-Ho Kang; Experiment: SukJin Kim, GaYoung Won; Co-wrote the paper: Soo-Im Choi, Sohyeon You; and Review paper: Gun-Hee Kim. All authors have reviewed and approved the final version of the manuscript.

## Conflicts of interest

The authors declare no potential conflict of interest.
